# Causal Inference Based on the Analysis of Events of Relations for Non-stationary Variables

**DOI:** 10.1038/srep29192

**Published:** 2016-07-08

**Authors:** Yu Yin, Dezhong Yao

**Affiliations:** 1 Key Laboratory for NeuroInformation of Ministry of Education, Center for Information in Medicine, University of Electronic Science and Technology of China, Chengdu, 610054, China

## Abstract

The main concept behind causality involves both statistical conditions and temporal relations. However, current approaches to causal inference, focusing on the probability vs. conditional probability contrast, are based on model functions or parametric estimation. These approaches are not appropriate when addressing non-stationary variables. In this work, we propose a causal inference approach based on the analysis of Events of Relations (CER). CER focuses on the temporal delay relation between cause and effect, and a binomial test is established to determine whether an “event of relation” with a non-zero delay is significantly different from one with zero delay. Because CER avoids parameter estimation of non-stationary variables *per se*, the method can be applied to both stationary and non-stationary signals.

Causality is a major concern in the study of complex systems; the detection and quantification of causal relations motivate many studies in various science domains. The foundation and connotation of causality have been continuously investigated for centuries by philosophers and scientists^1^,^2^,^3^,^4^, and the main concepts are rooted in probabilistic theories^5^. A mathematically and statistically general definition of causality given by Wiener involves two facts^6^: 1. the statistical condition, where *Y* causes *X*, indicating that the corresponding probabilistic dependencies obtain, that is, *P*(*X*) ≠ *P*(*X*|*Y*), and 2. the temporal relations, where only the past and present may cause the future but the future cannot cause the past. However, it is difficult to apply this formula in practice for two reasons. First, it is difficult to obtain *P*(X) without the effect of *Y* under natural conditions. This requires that experiments be conducted under highly controlled conditions. Therefore, we cannot determine whether the probability estimated from the time series *X* is *P*(X) or *P*(*X|Y*). Confidence is another issue, that is, whether or not *P*(*X|Y*) is significantly different from *P*(X) has to be considered.

Granger causality (GC)^7^, which came from Wiener’s definition of causality, and its derivatives^8^,^9^,^10^,^11^,^12^ have been widely used in recent decades. GC used the prediction to infer the causality. The prediction accuracy of a joint model that includes “cause” and “effect” is better than a model of “effect” alone. Another familiar approach is transfer entropy (TE)^13^, which depends on the overlap and dynamics of information transport by estimated probability distribution. However, it has been shown that TE and GC are equivalent for Gaussian variables^14^, and asymptotically equivalent for general Markov models^15^. Also, information-theoretic measures often require substantially more data than regression methods such as GC^16^. Recently, Pearl suggested that the laws of probability theory do not dictate how one property of a distribution changes when another property is modified; therefore, causal mechanisms should be stated in frame of causal inference^17^. He proposed a structural causal model^18^, which has received considerable attention in machine learning^19^ and artificial intelligence^20^. Most importantly, all of these approaches focus on probabilistic dependencies. They may involve linear models or information theory, and the performance usually depends on the parameters estimation^21^,^22^. Furthermore, for widely existed non-stationary variables such as various physiological signals, the corresponding parameters estimation is still an open question^23^,^24^. To avoid complex parameter estimation, this work focuses on the second fact of causality, the temporal relations.

Following the causality definitions of Wiener and Pearl, we propose a new model of causality that “effect *X*” will occur after a certain lag *τ* when “cause *Y*” happens. Suppose that *F*_*τ*_(*X*,*Y*) is joint probability distribution of *X* and *Y*, *τ* means *Y* at the earlier time. If *Y* doesn’t cause *X*, *F*_*τ*_ will not be significantly different from others. In contrast, if *Y* causes *X* in a lag *τ*′, the corresponding *F*_*τ′*_ would significantly differ from another *F*_*τ*_ such as *F*_0_. In this model, we assume that the sampling rate of data is high enough so that *τ*′ is not 0, because no information transmission is instantaneous. Thus, *F*_*τ*_ is compared with *F*_0_ to examine possible causality. To simplify the comparison, we suppose that *X* and *Y* are binary, only {*X* = 1|*Y* = 1} is concerned, which means “cause *Y*” and “effect *X*” both happened. Simply take *M*_*τ*_(*τ* ≥ 0) to note the event of relation (ER), which means that *X* and *Y* are observed, where *Y* is delayed by *τ*. *K*_*τ*_(*τ* ≥ 0) is the number of ER during the observation, and *p*(*M*_*τ*_) is the probability of those ER. Thus comparison is degenerated to a binomial test, and Poisson test (approximation of binomial test) is employed to simplify calculation (see Methods).

## Results

**Figure 1:**
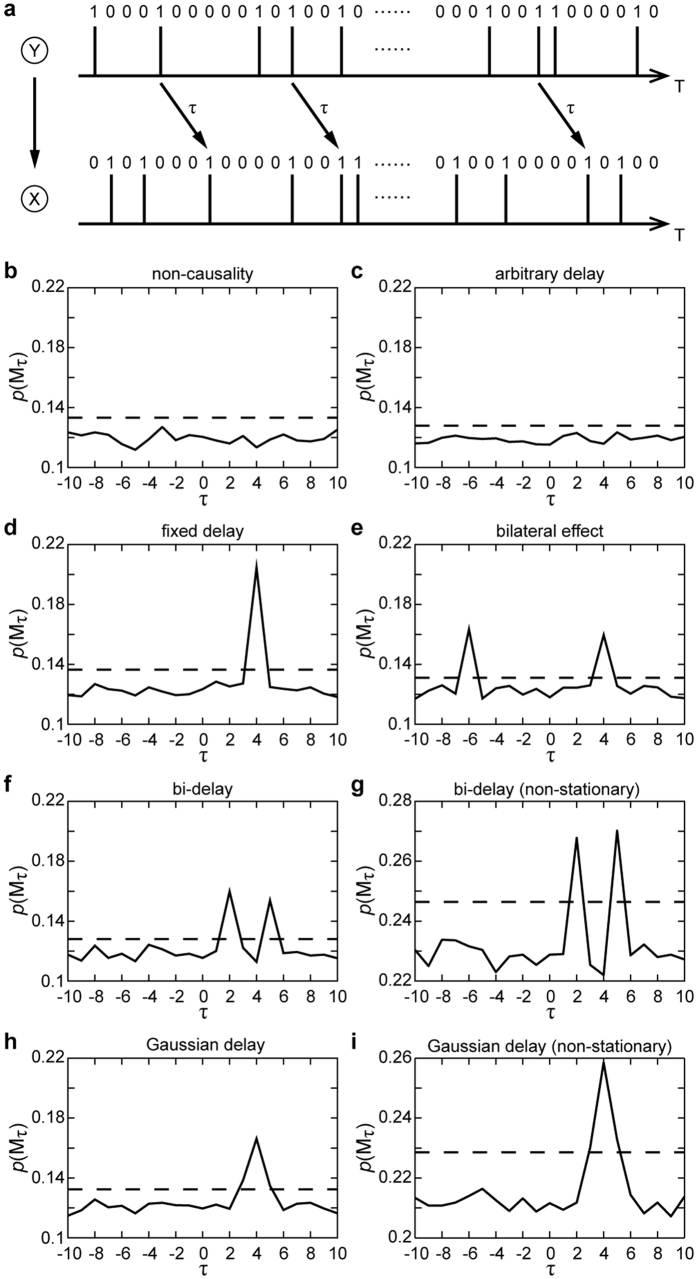
CER in discrete binary models. (**a**) Schematic of the causality of a two-node binary system. In following simulations, the event probability of the stationary series is fixed at 0.3; interactive probability *p*_*YX*_ = 0.6, except as specifically stated. (**b**) Non-causality: *p*_*YX*_ = 0, (**c**) Arbitrary delay: *τ* follows a uniform distribution (0 < *τ* ≤ 100). (**d**) Fixed delay: *τ* = 4. (**e**) Bilateral effect: *p*_*YX*_ = 0.3, *τ* = 4; *p*_*YX*_ = 0.3, *τ* = 6; no reciprocal action. (**f**) Bi-delay: *τ* = {2,5} with the same probability 0.5. (**g**) Bi-delay in non-stationary series. (**h**) Gaussian delay: using *τ* = {3, 4, 5} with probabilities {0.225, 0.55, 0.225}, respectively, to simulate a Gaussian distribution. (**i**) Gaussian delay in non-stationary series. The horizontal axis shows the delay time, and the vertical axis shows the probability (frequency) of ER. The dotted line is the upper limit by delay 0 at α = 0.005. The length of the simulation dataset is 5,000 points.

To test the efficiency of the CER, our causal inference approach based on the analysis of ER *M*_*τ*_(*τ* ≥ 0), the neuronal spikes interaction model^25^ with delay was simulated. In basic two-node interaction system based on the neuronal spikes interaction model, each node is driven by an event probability *p* to generate the binary time series. When node *Y* = 1, node *X* will be “1” with the interactive probability *p*_*xy*_ after a delay *τ* (schematic in Fig. 1a). The time series may be stationary or non-stationary depending on whether event probability *p* is constant or time-varying.

CER was tested for different types of lag and both stationary and non-stationary simulations. Figure 1b shows that *K*_*τ*_ of *M*_*τ*_ for different lags were not significantly different from 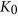 when the two stationary nodes were assumed to be non-causal. As Fig. 1c depicts the delay was a uniform distribution from (0, 100], which means that the “effect” can occur at any time after the “cause” appears. And *p*(*M*_*τ*_) was not higher than the upper limit, indicating that the Poisson test failed to reject the null hypothesis that *p*(*M*_0_) and *p*(*M*_*τ*_) are equal. In practice, the delay between two events having a causal relation is smaller than a certain value. Thus if the delay follows an unusually wide uniform distribution, it might be logically assumed to be non-causal. In contrast, Fig. 1(d–i) illustrate that the CER detected all pre-designed causality at accurate delay times under other conditions.

**Figure 2:**
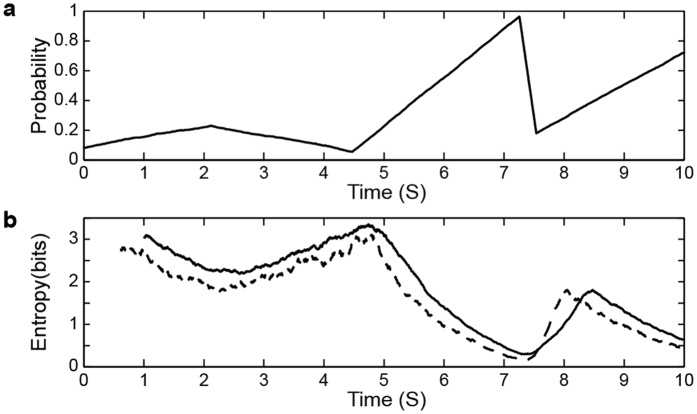
An example of non-stationary data. (**a**) Time-varying event probability; (**b**) Time-varying entropy of event series calculated with two different moving window lengths. Solid line: window length 1000 points, dotted line: window length 500 points. The data length is 10 seconds with a sampling rate of 1000 per sec.

Figure 2a illustrates a randomly generated (random walk) series of non-stationary probability *p* used to generate a binary time series to simulate non-stable spontaneous activity in one node. The non-stationarity of those time-varying *p* series was verified by Dickey-Fuller test (*P* < *0.05*) in all simulations. Two moving windows of different length were employed to calculate entropy of the non-stationary binary time series, respectively (Fig. 2b). The results indicated that the moving window technique might not be efficient for non-stationary data.

**Figure 3:**
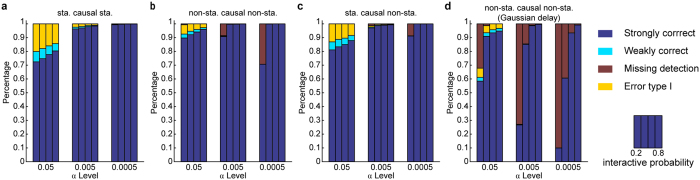
CER computational stability of a two-node system with different interaction probabilities at different test levels α. In each sub-graph, the statistical test at three α levels of 0.05, 0.005 and 0.0005 are shown in three bar graph groups. For each α level, the four bars are the results of using four interaction probabilities, 0.2, 0.4, 0.6 and 0.8. The total parameters are the same as in Fig. 1. Fixed delay (**a**–**c**): *τ* = 4. Gaussian delay (**d**): *τ* = {2, 3, 4, 5, 6} with probabilities {0.015, 0.21, 0.55, 0.21, 0.015}, respectively. The sta. denotes stationary variables, and non-sta. denotes non-stationary variables.

Detection rate and false-positive rate are two critical issues in a causal inference test, and five possible outcomes might be encountered in practice: 1. reject the null hypothesis at the right time (strongly correct); 2. reject the null hypothesis in the right causal direction without a well-defined delay (weakly correct); 3. failure to reject the null hypothesis when the variables are causally related (missing detection); 4. reject the null hypothesis but the causal direction is wrong (error type Ι); and 5. reject the null hypothesis but the variables are not causally related (error type ΙΙ). In our study the ratios of the five possible outcomes were calculated using 10000 runs of the numerical simulation and different simulation data were tested at three *α* levels (Fig. 3). The error type ΙΙ was almost zero (<0.7% at *α* of 0.0005) in all of these simulations, which are thus not shown. As Fig. 3 displays, the CER dominantly pointed to “strongly correct” outcome. The missing detection cases occurred mainly for data with weak interaction. Therefore, the CER exhibited a good performance in terms of excluding non-causality data with few errors. The error type Ι occurred mainly at *α* level of 0.05. At a more stringent *α* of 0.005 or even 0.0005, the ratio of the error type Ι decreased to nearly zero, and therefore, we may choose a smaller *α* when applying the CER.

Specifically, the detection rate could still be 100%, even for a non-stationary and Gaussian-distributed delay case (Fig. 3d) if the size of the dataset was sufficiently large and if the interaction was not overly low. Under this condition, *p*(*Y*_*τ*_*X*) may be significantly different from *p*(*Y*_0_*X*) at more than one delay *τ* (Fig. 1i). Such type of data is a substantial challenge for hypothesis testing, and the CER maintains high performance in this case.

**Figure 4:**
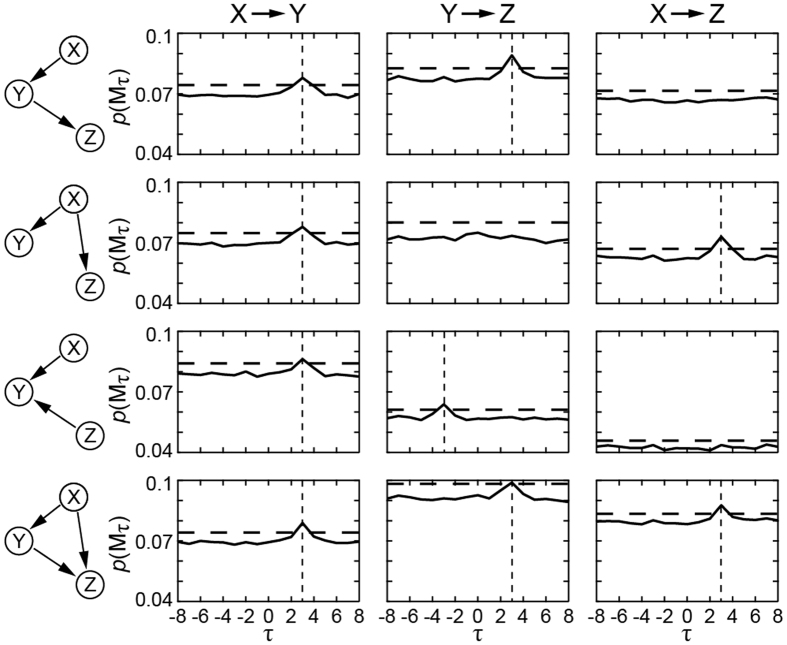
CER in DAG models of three nodes. The first column shows interaction of three nodes. The other columns are CER for different node pairs. The event probability is non-stationary; total interactive probability *p* = 0.6. Gaussian delay: *τ* = {2, 3, 4} with probabilities {0.225, 0.55, 0.225}, respectively. The horizontal axis shows the delay time, and the vertical axis shows the probability (frequency) of ER. The dotted line is the upper limit by delay 0 at α = 0.005. Note that there is a negative delay time because the direction of causality is tested for node *Y* to *Z* in the third column.

Now, we consider a possible complex case, directed acyclic graph (DAG). In DAG, nodes can be indirectly relevant to each other or respond to a common input^26^. The simulation of DAG was basically the same as the interaction model we previously used except that the node number was three instead of two. As the statistics we investigated are the temporal relations, inferring the direction of causality in DAG can be realized without knowledge of interested third-party. Therefore, the CER examined nodes in pair. It detected all pre-designed causality at an accurate delay time (Fig. 4).

## Discussion

One merit of the CER is the ability to process non-linear and non-stationary variables because it is based on the statistic variable “ER”, which does not depend on the dynamic process of variables *per se*. Moreover, causal inference may be greatly impacted by the performance of prediction models when GC and its derivatives are applied. In a complex system, the dynamic process of nodes may be totally different, and a joint model would be difficult to construct because of heterogeneous properties (e.g., being non-stationary or nonlinear). A copula approach^27^,^28^ has recently been proposed to reveal nonlinear, non-stationary causality and deal with binary events^29^,^30^; however, the algorithm requires kernel estimation, and it is still a challenge.

The core of the CER is the temporal relations in the definition of causality. Temporal relations were also implicated in other approaches^31^. In the widely applied GC, they are embedded in using the order of the regressive model. Differently, Pearl introduced “*do*(*x*)” for setting *X* = *x* and called the mapping from *x* to *P*(*y*|*do*(*x*)) for all *x* the causal effect of *X* on *Y*^17^. The operator “*do*(*x*)” is used to emphasize the occurrence of “*x*”. The “*x*” that previously occurred changes the distribution of “*Y*”, which implies temporal relations in causality. But in most conditions the above-mentioned approaches only identify past and future. How long will the “effect” delay after “cause” occurs? Or what does a distribution of the lags follow? Such information would also be meaningful for causality inference. In the CER, the temporal relation is a crucial variable. Our approach may detect not only the causal relation but also the lag or lag distribution.

In general, a high sample rate allows temporal properties to be exposed in the data and utilized in causality. If the temporal resolution is low, which means that “cause” and “effect” occur at the same sampling time according to Eq. (7), *K*_0_ will be larger than all *K*_*τ*_ and unable to determine the direction when nodes are causally related. However, one can still determine whether causality exists because the sign of the inequality is true in statistics.

It is common to record discrete data in many studies. Although the values of variables are often numerous, not all of the values are important. In many practical cases, binary or multiple values are common. In addition, discrete events are often objective reflections of many phenomena, and defining a discrete event is typically a goal during data processing. Moreover, data discretization provides information to answer particular questions. For example, in the analysis of relations between the prices (continuous value) of stocks *A* and *B*, it is informative to identify whether the price variations (binary value) of stock *A* affect the price variations of stock *B* during a period of time.

In summary, the CER approach features temporal relations, one crucial aspect of causality, and uses them as the basis of causal inference. It can be applied to non-linear, non-stationary, and binary variables. In addition, the CER can also be extended to systems of multiple values to address other causal problems. In the future use of the approach, the third-party variables may be included to reduce error type ΙΙ. An easy-to-use Matlab tool about causal inference can be downloaded from http://www.neuro.uestc.edu.cn/CER.html.

## Methods

Let us briefly review the causality definition summarized by Granger^32^. “For ease of exposition, a universe is considered where all variables are measured just at prespecified time points at constant intervals *t* = 1, 2… When at time *n*, let all of the knowledge in the universe available at that time be denoted *Ω*_*n*_ and denote by *Ω*_*n*_ − *Y*_*n*_ this information except the values taken by a variable *Y*_*t*_ up to time *n*, where *Y*_*n*_ ∈ *Ω*_*n*_. Suppose that we are interested in the proposition that the variable *Y* causes the variable *X*. At time *n*, the value *X*_*n*+1_ will be a random variable and so can be characterized by probability statements Prob (*X*_*n*+1_ ∉ *A*) for a set *A*”. If *Y*_*n*_ causes 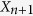, the following general definition can be suggested:


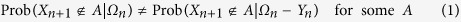


In this equation, the temporal relations are illustrated by a general delay indicated by the subscript “*n* + 1”. Here “+1” just means an interval to distinguish present between future.

But in the real world, the lag between “cause” and “effect” must be within a limited period. It might be a probability distribution or several discrete points. However, it would not be equal to zero because information transmission is not instantaneous. Therefore, the causality may be evaluated using the differences between a delay of zero and other options. We proposed that an important factor of causality is implicit stable temporal relations between variables. An appropriate statistical test and statistical variable are needed to confirm these differences. For simplicity, we assume that time series are binary “stochastic events”, i.e., the value of the variable is “1” when events have occurred and “0” when they have not occurred. We used *p* to denote the probability of the “stochastic event”.

In discrete cases, we can rename “stochastic variable” as “event”. Let us place events *X* and *Y* in the context of all of the knowledge in the universe; let *Ω* be a sample space of all measurable elements of interest in the universe; let *F* be a σ-field (collection of subsets of the *Ω*); and let *p* be a probability that is defined in measurable space (*Ω*, *F*), with *p*(*Ω*) = 1. The triple (*Ω*, *F*, *p*) is called a probability space, and events *X* and *Y* belong to the set *F*. The subscript *n* in Eq. (1) means that the past and present can cause the future, but the future cannot cause the past^31^. In time series, the time factor of causality is denoted by *τ*(*τ* > 0). Here, we employed time delay and binarization to depict event *Y* that previously occurred. As a result, for discrete events, Eq. (1) is still valid, and we can adapt it to:


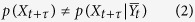


Here, 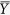 is the complementary set of *Y*. Event series *Y* shifted by *τ* along the timeline, denoted by *Y*_*τ*_. From here on, the subscript *t* is omitted for it is common for both *X* and *Y*. Such an operation does not change the probability of the occurrence of the event *Y*, i.e., *p*(*Y*) = *p*(*Y*_*τ*_). Then we adapt Eq. (2) to


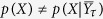



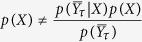



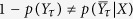


Because events *X* and *Y* belong to the set *F*, we have 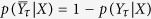; then,


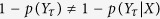



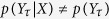



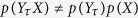



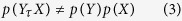


Since no information transmission is instantaneous, the *τ* is not equal to zero. If *τ* = 0 event *Y* does not cause event *X*. Thus Eq. (2) can be edited as follows:


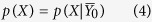


Following the same derivation from Eq. (2) to Eq. (3); from Eq. (4), we have


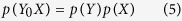


Next, merge Eq. (3) and Eq. (5) as follows:


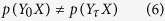


Eq. (6) illustrates that the joint probability *p*(*XY*) of *X* and *Y*, observed without delay, is different from that of *X* and *Y*_*τ*_ if *Y* causes *X* with delay *τ*. Conversely, if *X* and *Y* have no causal relation, then the joint probabilities *p*(*Y*_0_*X*) and *p*(*Y*_*τ*_*X*) will not be significantly different regardless of the value of *τ*. This means that the change in the observation time of one event does not affect the joint probability *per se* or any other non-causal event. Let us assume that someone rolls a coin first and a dice later. The non-causality of the experiments will not be changed if the person rolls a dice in one hour or even tomorrow. Hence, if the relation between events *X* and *Y* is not causal, then events *X* and *Y*_*τ*_ are not causal.

Now, let us denote the joint event of *Y*_*τ*_ and *X* as a new statistical event *M*_*τ*_, event of relation (ER), which means that events *X* and *Y* are observed, where *Y* is delayed by *τ*. As a result, Eq. (6) can be rewritten in terms of event *M*:


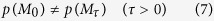


In this equation, “*Y* and *X*” are considered to be one object *M*, which is decided by the relations of events *X* and *Y* and not by *X* or *Y per se*. Hence, it is no longer necessary to know the probability of *X* or *Y*. Even if *X* and *Y* are non-stationary variables, the causal inference of ER can still be evaluated because it is investigated by the new statistical variable ER instead of the original variables.

In Eq. (7), *p*(*M*_0_) is compared to *p*(*M*_*τ*_) with different delays to determine whether the sign of the inequality is true. For binary series *X* and *Y*, only {*X* = 1 |*Y* = 1} is concerned, which means “cause *Y*” and “effect *X*” both happened; thus the ER values are 1 (cause and effect both happened) and 0 (other conditions). Therefore, a binomial test can be employed. The null hypothesis of Eq. (7) is that two probabilities are equal likely to occur. *p*(*M*_0_) is assumed to be the expected probability under the condition of non-causality. If the frequency of the ER observed with delay *τ* is significantly higher or lower than *p*(*M*_0_), then causality existed between the series data *X* and *Y*.

In the computation, we adopted a Poisson distribution, which is an effective approximation of a binomial distribution and easy to compute if the dataset is sufficiently large. When the number of observations is *n*, event *M*_0_ occurs *K*_0_ times, and event *M*_*τ*_ occurs *K*_*τ*_ times. If *Poission*(*K*_*τ*_, *K*_0_) > 1 − *α*, *K*_*τ*_ is believed to be significantly greater than *K*_0_ at the *α* significance level. In general, the “cause” is considered to increase the probability of the “effect”. If the “cause” decreases the probability of the “effect”, then it is called negative causality. Under this condition, *α* < *Poisson (K*_*τ*_, *K*_0_) can be used to determine whether *K*_*τ*_ is significantly smaller than *K*_0_. Apparently, both increase and decrease are processed in the same manner, except for the hypothesis test (i.e., right tail, left tail or two-tailed, respectively for increase, decrease or unknown cases). In this paper, only the increase case is considered in the simulations.

The algorithm of CER for binary series is summarized as follows:

1. Calculate the number of ERs with delay 0, *K*_0_ = *sum*{*X* = 1|*Y* = 1};

2. Calculate the numbers of ERs with different delay *τ*, *K*_*τ*_ = *sum*{*X* = 1|*Y* = 1}; *Y*_*τ*_ means series *Y* delayed by *τ*;

3. The Poisson cumulative distribution function of ER is given by


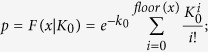


4. If *p*_*τ*_ = *F*(*K*_*τ*_|*K*_0_) < *α* indicates that *K*_*τ*_ is significantly smaller than *K*_0_; then *Y* has negative causality with *X* after delay *τ*;

5. If *p*_*τ*_ = *F*(*K*_*τ*_|*K*_0_) > 1 − *α* indicates that *K*_*τ*_ is significantly bigger than *K*_0_; then *Y* has positive causality *X* after delay *τ*.

## Additional Information

**How to cite this article**: Yin, Y. and Yao, D. Causal Inference Based on the Analysis of Events of Relations for Non-stationary Variables. *Sci. Rep.* **6**, 29192; doi: 10.1038/srep29192 (2016).
